# Supplementary material for: Patterns of Intron Gain and Loss in Fungi
Source: PLoS Biol. 2004 Nov 30;2(12):e422. doi: 10.1371/journal.pbio.0020422 (PMC532390; doi:10.1371/journal.pbio.0020422)
Supplement: Table S1 — Also available at http://genes.mit.edu/NielsenEtAl/. (4.3 MB ZIP). [file pbio.0020422.st001.zip › NielsenEtAl/html/948.html]

AN1949.1.NCU09349.1.MG06522.1.FG04350.1


```
 CLUSTAL W (1.82) Multiple Sequence Alignments - Introns Inserted


Sequence 1: MG06522.1	578 aa
Sequence 2: FG04350.1	590 aa
Sequence 3: NCU09349.1	578 aa
Sequence 4: AN1949.1	609 aa
Alignment Length: 610 aa
Number Identitical Residues: 359 aa
Alignment Score (without introns) 15160


MG06522.1 	------MDDRSSKKRKLKD---VNGAKASGAATDVTVKPKKLKKAQSQE-EVEPK-VEKG
NCU09349.1	------MASEFSKKRKLKD---AKIATEDGAATDK--KTKKVKKDKKEKKAAEEV-VEDA
FG04350.1 	------MDFASSKKRKFKD---ANGVKPSKGKKSSSIPDKKSKKVKRAEPEPHDE-PEDD
AN1949.1  	MPAAVDTAKTISKKRKRKHGGSAREATDNGVATSPAENGVEGKATETTTELTKDKDVELA
          	 .:: .     ***** *...:.. .. .   .. :    : *  :      .  . *  

MG06522.1 	SKKEEEENDWSDEEENEAADDAGHSSDSDVDEGDDTIAAPAKEAD----GDIP--GDLTL
NCU09349.1	TPEEENDAQKAEEQQD--GDEVIPTGNGDKEDSDD----KDAEAG----DELTKTNDSLI
FG04350.1 	SSDEEEQALKEVEDESDQADEDVEAANSAEEEEEEGGDEDNAENN----TDLPNGGQLTL
AN1949.1  	KKVVKEEAQKKRKVSHSPSDEEESESEDEEKKEEDEKDNVKEDSGKANVPDLPSVDAVSL
          	.   :::     : . . .*:    .:.  .. ::       : ..:.  ::.. .   :

MG06522.1 	PTTAESEAQAFSELNLSENTMKAIEEMGFTKMTEIQRRGIPPLLAGKDVLGAAKTGSGKT
NCU09349.1	APSIATNATDFSELNLSDKTMKAIAEMGFTKMTEIQRRGIPPLLAGKDVLGAAKTGSGKT
FG04350.1 	PPVAGAEAQSFEELKLSEKTMKAINEMKFTKMTEIQRRGIPPSLAGRDVLGAAKTGSGKT
AN1949.1  	P-QPDGGPKKFTELGLSEKTLQGIKEMGFETMTEIQQRTIPPLLAGRDVLGAAKTGSGKT
          	.      .  * ** **::*::.* ** * .*****:* *** ***:*************

MG06522.1 	LAFLIPAVEMLRSLKFKPRNG~TGVIVVSPTRELALQIFGVARDLMKHHSQTYGIVIGGA
NCU09349.1	LAFLIPAIEMLSSLRFKPRNG~TGAIVVTPTRELALQIFGVARELMKNHSQTYGVVIGGA
FG04350.1 	LAFLIPVIEMLSSLRFKPRNG~TGVIVVSPTRELALQIFGVARELMAHHSQTYGIVIGGA
AN1949.1  	LAFLIPAIEMLSALRFKPRNG1TGVIVVSPTRELALQIFGVARELLTAHSQTYGIVIGGA
          	******.:*** :*:****** **.***:**************:*:  ******:*****

MG06522.1 	NRRAEAEKLSKGVNLLIATPGRLLDHLQNTP-FVFKNLRSLVIDEADRILEIGFEDEMRQ
NCU09349.1	NRRAEAEKLGKGVNLLIATPGRLLDHLQNTP-FVFKNMRSLIIDEADRILEIGFEDEMRQ
FG04350.1 	NRRAEAEKLAKGVNLLIATPGRLLDHLQNTP-FVFKNLKSLVIDEADRILEIGFEDEMRQ
AN1949.1  	NRRAEAEKLTKGVNLLIATPGRLLDHLQNTPGFVFKNLKTLVIDEADRILEVGFEDEMRQ
          	********* *********************.*****:::*:*********:********

MG06522.1 	IIKILPKE-RQSMLFSATQTTKVEDLARVSLRPGPLYLNVDEEKEYSTVEGLEQGYVVCE
NCU09349.1	IIKILPKEDRQTMLFSATQTTKVEDLARISLRPGPLYVNVDEEKQFSTVEGLDQGYVVVD
FG04350.1 	IIKVLPKEDRQTMLFSATQTTKVEDLARISLRPGPLYINVDEEKQYSTVEGLEQGYIICE
AN1949.1  	IIKILPNEDRQTMLFSATQTTKVEDLARISLRPGPLYINVDHRKEHSTVEGLEQGYVICE
          	***:**:*.**:****************:********:***..*:.******:***:: :

MG06522.1 	ADKRFILLFSFLQKMKKKKIIVFFSSCNSVKYYAELLNYIDCQVLDLHGKQKQQKRTNTF
NCU09349.1	ADKRFLLLFSFLKKMQKKKVIVFFSSCNSVKYYSELLQYIDLQVLDLHGKQKQQKRTNTF
FG04350.1 	TDMRFLLLFSFLKRNLKKKIIVFFSSCACVKYHAELLNYIDLPVLDLHGKQKQQKRTNTF
AN1949.1  	ADKRFLLLFSFLKRNLKKKIIVFFSSCNCVKYHAELLNYIDLPVLDLHGKQKQQKRTNTF
          	:* **:******::  ***:******* .***::***:***  *****************

MG06522.1 	FEFCNADRGTLICTDVAARGLD0---------FDPPDDPRDYIHRVGRTARGTNKKGRSL
NCU09349.1	FEFCNAKQGTLICTDVAARGLD~IPAVDWIVQFDPPDDPRDYIHRVGRTARGNNTKGRSL
FG04350.1 	FEFCNAKQGTLICTDVAARGLD0IPSVDWIIQFDPPDDPRDYIHRVGRTARGSNNKGRSL
AN1949.1  	FEFCNAKQGVLICTDVAARGLD0IPAVDWIIQFDPPDDTRDYIHRVGRTARGANGRGRSL
          	******.:*.************  .: .   .******.************* * :****

MG06522.1 	MFLLPSEVGFLTYLKQARVPVVEFDFPTKSIKNVQSQLEKLIGKNYYLNS~SAKDGFRSY
NCU09349.1	LFLQPNELGFLAHLKAAKVPVVEYDFPKSKILNVQSQLEKLIGQNYYLNQ~SAKDGYRSY
FG04350.1 	MFLQPNELGFLSHLKAARVPVAEFNFPTKKIINVQSQLEKLISQNYYLNK~SAKDGYRSY
AN1949.1  	MFLQPSEVGFLKYLKEARVPVVEFDFPAKKIVNVQSQLEKLISQNYYLNK0SAKDGYRSY
          	:** *.*:*** :** *:***.*::** ..* **********.:*****. *****:***

MG06522.1 	LHAYASHSLRSVFDINKLDLAKVAK~SFGFATPPRVDIQLGASMSKDKKAG--GRRAYGS
NCU09349.1	LHAYASHSLRSVFDIHKLDLVKVAK0SFGFSTPPRVDITLASSMSRDKKQT--SRRAYGS
FG04350.1 	MHAYASHSLRSVFDINKLDLAKVAK~SFGFTQPPRVDITLGASMSRDKKQQ--GRRAYGS
AN1949.1  	LQAYASHSLRSVFDVHKLDLVKVAK~SFGFSTPPRIDIQLGSSLSRDKKQQQQGRRSYGS
          	::************::****.**** ****: ***:** *.:*:*:***  ...**:***

MG06522.1 	QPRQGGT~YGKRR--
NCU09349.1	QPKQNRH~-------
FG04350.1 	QPRQNQA1AAGS---
AN1949.1  	QPKGLKF~KRKHEDD
          	**:          ..
```
